# Development and Content Validation of the Swimming Competence Assessment Scale (SCAS): A Modified Delphi Study

**DOI:** 10.1177/00315125231177403

**Published:** 2023-05-18

**Authors:** Jon Sundan, Monika Haga, Håvard Lorås

**Affiliations:** 1 Department of Teacher Education, 8018 Norwegian University of Science and Technology, Trondheim, Norway

**Keywords:** children’s aquatic competence, swimming skills, sport assessment, water safety, sport psychometrics

## Abstract

The purpose of this study was to design and develop the Swimming Competence Assessment Scale (SCAS) to measure children’s aquatic skills as they align with the physical education curriculum for Norwegian primary schools. We conducted a three-round modified Delphi study involving 22 national experts in the aquatic profession. Experts reached consensus on scale items within an observation form and coding sheet based on a swimming proficiency test for measuring six aquatic skills: water entry, frontstroke swimming, surface dive, float/rest, backstroke swimming and water exit. Independent experts obtained high agreement (scale level: 88%, item level: 80–93%) on the relevance, representativeness, and clarity of the scale. Current results suggest that the SCAS is a valid instrument for researchers and practitioners to observe and record children’s aquatic proficiency for the purpose of screening and developing aquatic education.

## Introduction

Many children seem to appreciate the vivid playground that recreational aquatics represent, with their capacity for promoting joy of movement and physical activity throughout the life span. The acquisition of swimming skills has received increased research attention due to safety risks for children associated with activities in and around aquatic environments worldwide (Bierens, 2014; Brenner et al., 2003; World Health Organization, 2014). As a precondition for safe engagement in aquatics, the World Health Organization (WHO) has strongly recommended that children (age six or older) from all countries be empowered with basic swimming and water safety skills through educational programs (World Health Organization, 2017; 2021; 2022).

While several countries have embedded swimming and water safety training into their national educational curriculum, there appears to be a large difference in how nations, states or regions emphasize school based swimming and water safety activities, and how they define the ability to swim (United Nations Educational Scientific and Cultural Organization, 2014). In Norway, teaching basic swimming skills to children has been a statutory requirement since the first national school plan in 1939 (Ministry of Church and Education, 1957, p. 194), and in-school swimming and water safety have since been mandated within the physical education (PE) curriculum. Several aims for students’ swimming competence are stated throughout the 10-year compulsory education period, ranging from being water confident in early school years to performing more comprehensive swimming and water safety skills in later years. In 2015, a new (and present) standard for swimming proficiency (herein referred to as swimming competence) was introduced. The previous swimming competence standards utilized a traditional approach that emphasized the four competitive strokes and an arbitrary requirement of being able to cross 25–200 m of deep water by any means of self-propulsion (Bierens, 2014, p. 199; Department for Education, 2013, p. 200; Finish Swimming Teaching and Association, 2011; Moran, 2013; Royal Life Saving Society Australia, 2019, p. 11). The new standard focus on a more diverse and comprehensive objective that includes performing six consecutive aquatic skills that anticipate the skill acquisition level expected of a 9–10 year old child (fourth grade), as stated in the Norwegian PE curriculum:

“be able to swim by falling into deep water, swim 100 m frontstroke, surface dive and pick up an object with your hands during swimming, stop and rest for 3 min (while floating on your front, orienting yourself, rolling over, and floating on your back), then swim 100 m backstroke and get ashore” (Norwegian Directorate for Education and Training, 2020, p. 6).

The latest PE curriculum revision (Norwegian Directorate for Education and Training, 2020) presented a shift in general perspective, wherein a less sports-oriented approach and holistic view to PE was suggested (Bratten & Kilanowska, 2021). This shift implies that swimming is not reduced to merely propulsion and “moving forward,” but emphasize a broad repertoire of aquatic skills. This change in focus closely relates to the *water competence* construct (Langendorfer & Bruya, 1995), that promotes a broad, all-around development of aquatic skills. Additionally, knowledge, attitudes, and values from a range of aquatic environments and subdisciplines must be developed to be water competent (Button et al., 2020; Quan et al., 2015; Stallman et al., 2017). Stallman (2017) argued for a paradigm shift in aquatics from swimming skills to water competence in which the goal is to prevent drowning rather than to develop competitive swimmers.

In 2017, the Norwegian government established a compulsory swimming proficiency test to be completed before the end of fourth grade (Ministry of Education and Research, 2021). The proficiency test aims to monitor the achievement of learning outcomes (i.e. the children’s progression towards being a competent swimmer). Several studies have focused on an assessment of aquatic skills through the use of various test instruments (see for example Wizer et al., 2021 for an overview). However, existing tests have not been suited for measuring swimming competence in a Norwegian context, due to the specific fourth grade learning objective in the Norwegian PE curriculum. Some tests have been targeted to other age groups (Erbaugh, 1978; Mertens et al., 2022; Moreno-Murcia et al., 2020), while others have focused on specific tasks or a series of non-consecutive aquatic tasks (Kjendlie et al., 2013; Moreno-Murcia, 2005). While two studies have targeted children’s swimming proficiency levels in the Norwegian context (Mordal Moen et al., 2018; Norwegian Swimming Federation, 2021), both rely on limited self-reported estimates of the students’ own capability, and one explored swimming competence only in terms of distance covered.

As evidenced by the literature discussed above, there is a need for a new standardized swimming competence instrument for children and youth (Button, 2016). Such an assessment would allow us to obtain knowledge about the levels and progress of aquatic learning among school-aged children and further enhance our evaluation and development of swimming education programs. We employed a modified Delphi study to develop a valid and practical instrument for researchers and practitioners to use when measuring swimming competence in 9–10-year old schoolchildren. The aims of this instrument were: (a) to observe and score basic aquatic skills; (b) to guide pedagogical development in learn-to-swim programs (i.e., become a source for formative assessment of students, parents/legal guardians, and teachers); and (c) be a useful instrument for further research and development purposes. The scope of the study was predetermined by the specific fourth grade learning objective in the Norwegian PE curriculum, requiring that a 9–10 year old child must master six consecutive aquatic skills to be characterized as swimming competent: (a) deep water entry, (b) 100-m forward swimming, (c) surface dive, (d) 3 min floating/resting, (e) 100-m swimming on back, and (f) water exit.

## Method

### Participants

We recruited 22 swimming expert participants (15 males, seven females; *M* age = 49.8, *SD* = 12.4) from the national aquatic profession (Table 1). After volunteering and providing informed consent, the participants were directed to the task force group (TFG) or the independent expert panel (IEP) subgroups, based on their practical experience, educational backgrounds, and institutional affiliations. Experts were recruited through targeted sampling, and they had to meet the following inclusion criteria: (a) specialization within the topics of swimming and water safety, (b) extensive experience and affiliation with learn-to-swim programs for children, and (c) knowledge about the Norwegian PE curriculum. In our recruitment efforts, we sought participants from a range of disciplines (e.g., academic expertise, swimming instructors, PE teachers or swimming federation representatives) to gather a broad understanding of swimming competence. This study was conducted in accordance with the guidelines from the National Committee for Research Ethics in Social Sciences and the Humanities, and it was accepted by the Norwegian Centre for Research Data (NSD).

**Table 1.** Characteristics of Expert Panel Members.

Characteristics	TFG (*n* = 7)	IEP (*n* = 15)	Total (*n* = 22)
Gender (male/female)	6/1	9/6	15/7
Age (years)	39.3 (29.6–55.2)	53.5 (38.8–82.1)	48.9 (29.6–82.1)
Educational level			
Bachelor	2	2	4
Master	3	5	8
PhD or equivalent	—	7	7
Other	2	1	3
Institutional affiliation			
University	1	11	12
Primary school teacher	2	2	4
Swimming instructor	3	1	4
Norwegian Swimming fed	1	1	2
Scientific publications			
None	6	9	15
1–3	1	3	4
>4	—	3	3
Years of experience	14.9 (7.8–27.4)	27.0 (7.7–55.3)	23.2 (7.7–55.3)

### Scale Development

The development process involved consensual guidance from experts on aquatic education, using a Delphi research method (Vernon, 2009). The Delphi approach allows “a group of individuals as a whole, to deal with a complex problem” (Linstone & Turoff, 1975, p. 3); it can be described as a structured communication process aimed at collecting knowledge and generating a consensus of expert opinion through the administration of a repeated series of surveys alternated with controlled opinion feedback (Ross et al., 2014). This method can be advantageous for optimizing the instrument’s ability to meet practical and pedagogical needs for both researchers and practitioners in aquatic learning processes. The process of selecting experts is important because the value of the process depends on the use of pooled expert knowledge and judgement to inform future decision making (Jünger et al., 2017; Ross et al., 2014).

**Figure 1.**
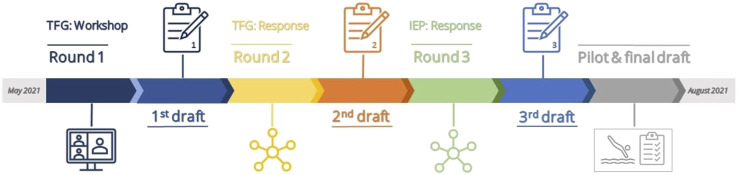
Measurement Instrument Development Process.

We established the development and content validation of the Swimming Competence Assessment Scale (SCAS) through a three-round modified Delphi technique of consensus building through iterative structures that were tailored and facilitated by the first author. We conducted the Delphi study from May to August 2021 (see Figure 1), focusing on the standardization of procedures for a swimming competency test, developing a practical observation scale with individual scores on each test item, and developing an accompanying coding sheet for data registration. Each round of the Delphi study ended with summarizing information and expert opinion feedback into a draft that, in turn, informed the design of subsequential Delphi rounds in which the participants could adjust their earlier responses.

This study used modifications to a traditional Delphi application, which normally contains a rigid design with rounds of questionnaires, polls, and rankings to establish a certain content. In this study, round one replaced the typical questionnaire with a participatory workshop, in which experts engaged in a stimulating exchange of their experiences in discussing the topic. This modification was arguably an exploratory approach that brought this process closer to a Responsive Delphi design, in which participants are presented with a previously generated defined list of topics or issues for comment (Vernon, 2009). This approach led to a partial loss of anonymity during the TFG, in contrast to the IEP, in which anonymity was preserved. Both approaches are common when they are perceived to be beneficial to the study objectives (Vernon, 2009). For example, anonymity can be advantageous when the researcher wishes to remove problems associated with face-to-face disagreements or bias, like wasting time or unhealthy group dynamics. For the TFG, we desired a dialog or discussion between participants, resulting in a face-to-face workshop.

### Round I Procedures

We initiated the development process by gathering the TFG of six other experts (6 males, one female; *M* age = 39.9, *SD* = 9.4), recruited from different professions in the field of aquatics. Representatives of the TFG participated in a face-to-face workshop, which, due to corona pandemic restrictions, was conducted on a digital platform (Zoom Meetings) in May 2021, and was recorded for later analyses. The first part of the workshop aimed to exchange expert information, experiences, and viewpoints on the assessment of swimming competence in children. The facilitator distributed a series of questions designed to elicit a structural brainstorming and allowed all participants in turn to elaborate on their perspectives of test procedures and scoring of children’s aquatic performances. The second part of the workshop was designed as a discussion forum, in which the facilitator pinpointed the experts’ contradictive or opposing opinions, striving towards establishing common ground and building consensus. Following the workshop, the facilitator finalized round one by collating and synthesizing information obtained into a first draft of the SCAS.

### Round II Procedures

In round two, the first draft of the SCAS was critically revised by the TFG. We began by providing the group with the documents describing the test procedures, the observation form and the coding sheet. The experts were given the opportunity to adjust their original answers through a series of prepared open-ended questions. Communication with the TFG in round two was conducted through individual email correspondence within a specific time frame, enabling the participants to give responses without social or conformity pressures to form a dominant view that emerged in round one. Based on responses from the TFG in round two, the SCAS was revised.

### Round III Procedures

In round three, the SCAS was distributed to 15 additional independent experts (9 males, six females; *M* age = 53,7, *SD* = 11.9), alongside a digital content validation form with instructions. The IEP consisted of a homogeneous group of specialists on aquatic education who had not earlier been involved in the scale development process. They were asked to critically review the observation form. The IEP members responded to a questionnaire containing 5-point Likert scale questions intended to be used to score the children’s performance on each of the six aquatic skills related to the relevance, representativeness, and clarity of the questions for measuring swimming competence in children. The Likert scale options were: (1) strongly agree, (2) agree, (3) neutral (ambivalent), (4) disagree, or (5) strongly disagree. The IEP members were also encouraged to provide written comments to give specific responses about the entire assessment instrument, and a potential justification of their opinions. This communication was conducted through individual e-mails, with anonymity between responders. Based on these responses, further minor adjustments were made to the SCAS.

### Pilot Study

Next, we conducted a pilot study in June 2021 to examine the application of the SCAS in a practical context. Volunteer participants for this pilot study were 12 children (6 boys and six girls) in the fourth grade of public primary school. Children and parents/legal guardians provided informed consent to participate in the pilot study. A swimming proficiency test applying the SCAS test procedure was conducted in a 12.5 m standard indoor swimming pool (depth 1.3 m), as a natural part in the end of the participants learn-to-swim education program in PE. The first author and one member of TFG (swimming instructor) organized, observed, and recorded data, and gathered other information about the practical aspects of the proficiency test. After the pilot study, we further revised the SCAS into a final version.

### Analysis and Interpretation

No a priori unanimous consensus definition of swimming competency was established. However, the TFG members defined the in-group consensus threshold that the main proportion (5 of 6) of participants agreed on particular viewpoints of this construct, which is a common definition of the type of consensus normally reached in Delphi studies (Diamond et al., 2014). All responses were taken into consideration and carefully reviewed to refine SCAS, and, subsequent to each round of discussions, responses were synthesized and categorized as either *high consensus* responses or *disagreements*. Agreement responses became the prime instigator in the process, whereas disagreement responses were either included or discarded, as guided by the participants’ majority views and opinions and their judgments of response suitability when contrasted with the relevant literature. Changes between rounds were labeled as major or minor alterations when tracking the scale development process, where the category of minor alterations consisted of small wording changes or changes in sentence structure or layout, and major alterations consisted of changes in content that contributed to a significant change in the measurement instrument.

To quantify the IEP members’ agreement, we used SPSS Statistics (version 28.0.1.) for statistical analyzes. The content validity index (CVI) is a widely reported approach (Polit & Beck, 2006; Yusoff, 2019), and in this study the CVI was computed as content validity at both item-level (I-CVI) and scale level (S-CVI/*Ave*). I-CVI reflected the proportion of the IEP scoring items on the 5-point Likert scale that had a relevance of one or 2 (Strongly agree or Agree). S-CVI/*Ave* Was computed by the sum of I-CVI scores divided by the number of items. Lynn (1986) described the content validity index scores as excellent when 78% agreement are obtained at item level, and 90% at scale-level.

## Results

The instrument produced through this consensus building with experts contained a scale for observing children’s swimming competence, a coding sheet for data recording, and procedures for conducting a standardized swimming proficiency test for children. The evolvement of consensus from each round, and the analysis of the content validity of scale are presented in the following sections.

### Round I Outcomes

The initial workshop revealed a high consensus among experts on the main intentions of the swimming proficiency test, to assess children’s swimming competence in line with the curricular aims and learning outcomes. The TGF suggested that the six different aquatic skills to be performed continuously in a swimming proficiency test should simulate a water submersion self-rescue situation (Stallman et al., 2008). The order of the described skills had a logical structure and progression from entering to exiting the water environment. However, TGF panelists recommended a more dynamic approach in addition to entry and exit skills, whereby the remaining four skills might occur in a variable order and be performed as appropriate for the test context. Moreover, the TFG members were explicit about the possibility of skipping one aquatic skill during the course of the test, if necessary, to still carry out the remaining skills to document as much of the children’s competence as possible. The TFG members finished round one by achieving consensus on fundamental aspects in the general procedures for the proficiency test (see Table 2).

**Table 2.** Test Procedure for Aquatic Skill Fundamentals.

Aquatic Skill	Test Procedure
Entry into water	• Entry with total submersion • Deep water to prevent footing when entering • Controlled resurfacing and orientation
Swim on front	• Level off and continuous progression by propulsion • Little focus on swimming technique or time used
Float/rest	• Aiming to rest and save energy • Horizontal body position suggested but not vital, if objective of lowered energy use is obtained
Surface dive	• Horizontal to vertical position • Pick up object (e.g., a diving ring) from pool floor to measure coping with depth, pressure and reduced visibility
Swim on back	• Level off and continuous progression by propulsion • Little focus on swimming technique or time use
Exit water	• Exit from deep water, without the use of feet when climbing • Option to simulate climbing onto a dock or other elevated surface

### Round II Outcomes

Due to high consensus on test procedure fundamentals in round one, the focus in round two shifted toward providing responses on the observation form and coding sheet, and establishing an updated version of the SCAS (Table 3). The observation form was suggested by the facilitator to contain five proficiency level scores for each aquatic skill to thoroughly judge the children’s aquatic behaviors within a range from high to low proficiency. Based on the test procedure fundamentals and proposed scoring system, a preliminary observation form and a coding sheet were established. However, to achieve the practical objective and simplify data recording for raters, the TFG members’ suggestion was to reduce this scoring system from five to four rating options: (1) very high, (2) high, (3) low, and (4) very low. These results are presented in sections providing an overview of essential changes, input, and additions to the preliminary draft for each aquatic skill.

**Table 3.** Aquatic Skills, Scores and Descriptions.

Aquatic Skill	Score	Description
Entry into water	4	Fall into deep water fully submersed, resurface and continues
	3	Fall into deep water from squat position fully submersed, resurfaces and continues
	2	Fall into deep water immersed or submersed, resurfaces, holds on to edge/swim lane lines or similar and continues
	1	Unable to fall into deep water and submersion, and enter by climbing
Swim on front	4	Swimming continuously 100 m frontstroke
	3	Swimming 100 m frontstroke, but has to stop and rest in the water one or more times along the way
	2	Swimming 100 m frontstroke, but has to rest standing on the pool floor, hanging on the edge, or pool lane lines
	1	Unable to swim 100 m frontstroke
Float/rest	4	Floats effortlessly for 3 minutes without significantly correction resting position
	3	Floats relatively effortlessly for 3 minutes, but corrects resting position repeatedly with active movements
	2	Floats strained for 3 minutes, and must work hard to be able to hold the resting position
	1	Unable to float for 3 minutes
Surface dive	4	Dives from the surface to the pool floor, and perform the task on first attempt
	3	Dives from the surface to the pool floor, and perform the task on second attempt
	2	Dives from the surface to the pool floor, but need three or more attempts to perform the task
	1	Unable to surface dive to the pool floor and perform the task
Swim on back	4	Swimming continuously 100 m backstroke
	3	Swimming 100 m backstroke, but has to stop and rest/float in the water one or more times along the way
	2	Swimming 100 m backstroke, but has to rest one or more times standing on the pool floor, hanging on the edge, or pool lane lines
	1	Unable to swim 100 m backstroke
Exit water	4	Exits the pool to an edge elevated above the water surface (e.g. 30 cm)
	3	Exits the pool to an edge horizontally with the water surface
	2	Exits the pool to an edge horizontally with the water surface, but needs several attempts
	1	Unable exit the pool without using a ladder/stair or other assistance

### Entry into Water

The TFG emphasized operationalizing the term *enter into water*, with a description of how entering the water should be performed. Merely jumping into the water could contrast to the self-recue perspective of the proficiency test, where the objective was to simulate an unintentional fall into deep water. Responses from the TFG resulted in a clarification that to gain a high (3) or very high (4) score, the child would be required to be fully submersed by falling into the water. However, several entry methods were possible (e.g., sideways, backwards, or with a consented soft push).

### Swim on Front and Back

A high and very high score on the scale refers to an aquatic performance that implies continuous progression in the propulsive phase, with no stopping to rest by holding on to the edge of the swimming pool, swim lane lines, or other supportive devices. The TGF added that, in test situations where unintended events occur (e.g., disturbance from peers or collisions), this continuous progression should be considered when scoring frontstroke and backstroke performances. Two of the panelists suggested to add sidestroke swimming to this category, but no consensus was attainted on this topic regardless of its relevance, and this proposition was discarded.

### Surface Dive

The TFG responded that a standardized depth should be implemented in the test procedures for the surface dive to avoid swimming pool construction becoming a factor when scoring underwater performance. High consensus was reached that a diving depth of 1.3 m would be necessary for 9–10 year old children, and this depth was implemented in the test procedures.

### Float/Rest

In the TFG responses, a high consensus was achieved about the importance of floating as an aquatic skill to rest and save energy. Furthermore, the degree of motionless horizontal position had to reflect the individual’s body composition in determining their floating capacity (e.g., muscle mass density and lowered buoyancy), involving the possibility to maneuver with minimal movements of the arms and legs. The TFG also emphasized that the individual should be allowed to change resting positions from back to front repeatedly, control their breathing and look effortless in their floating competence.

### Exit Water

The single response achieving high consensus amongst the TFG members regarding exiting water was that members described a standard height (30 cm) of the elevated edge for exiting the pool, eliminating variable heights to decide the level of competency displayed on the test.

In addition to the responses regarding the different aquatic skills, TFG proposed to establish a dichotomous category where the observers/raters subjectively determined if the child was able to swim or not by definition, which was implemented in the right row of the coding sheet for data recording (Table 4).

**Table 4.** Coding Sheet for Recording Data.

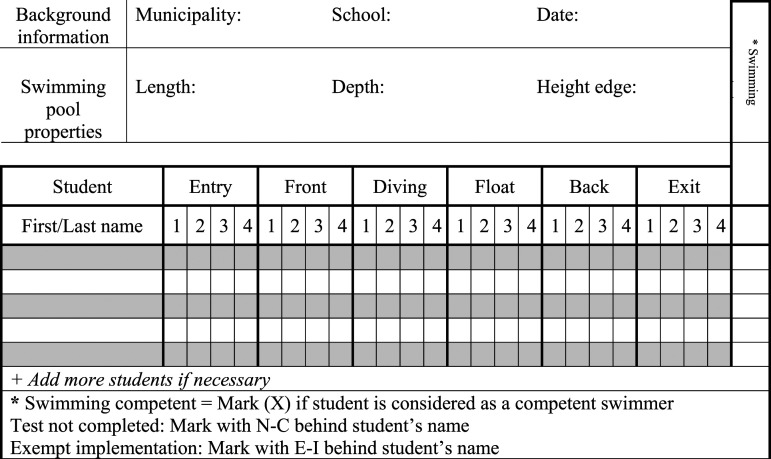

### Round III Outcomes

IEP responses on scale relevance are shown in Table 5, along with content validity index scores at both the item and scale level. I-CVI ranged from .80 to .93 on the six items, and S-CVI/*Ave* Was calculated to *.87*. S-CVI/UA is not reported due to a lack of agreement among experts at item level.

**Table 5.** IEP Content Validity Responses.

	Experts																
	1	2	3	4	5	6	7	8	9	10	11	12	13	14	15	No. of agreement	I-CVI
Entry	3	3	2	1	2	2	1	2	1	2	1	1	1	1	2	13	.93
Frontstroke	1	2	2	2	2	1	2	1	1	3	2	1	1	1	2	14	.93
Surface dive	1	4	3	2	2	2	1	1	1	4	1	1	2	1	1	12	.80
Float	1	3	2	1	2	1	2	1	1	3	1	1	2	1	1	13	.87
Backstroke	1	2	2	2	2	1	2	1	1	3	2	1	1	1	2	14	.93
Exit	2	2	3	1	2	2	3	2	1	2	2	1	1	1	1	13	.87
																S-CVI/Ave	.88

### Pilot Study

The objective of the pilot study was to rehearse the upcoming main study, with a focus on the scale’s feasibility. The pilot study resulted in minor adjustments to the test protocol (e.g., linguistic changes and modifications in communicating trial design to children in pre-testing situations).

## Discussion

By means of a modified Delphi study described here, we developed the Swimming Competence Assessment Scale (SCAS) to measure children’s aquatic capabilities. The backdrop was a specific competence aim in primary school for fourth grade (9–10 year old) children, that constitutes swimming competence in the Norwegian PE curriculum (Norwegian Directorate for Education and Training, 2020). Furthermore, it can be argued that SCAS operationalizes the psychomotor aspect of the water competence construct coined by Langendorfer and Bruya (1995), which, in terms of drowning prevention, is defined as the *“*sum of all personal aquatic movements that help prevent drowning, alongside water safety knowledges, attitudes, and behaviors that facilitate safety in, on and around water” (Moran, 2013, p. 4).

The Delphi process resulted in the development of an assessment scale and general procedures for conducting a swimming proficiency test, including an observation form and a coding sheet (see appendices) for six consecutive aquatic skills: (a) entry, (b) frontstroke, (c) surface dive, (d) float/rest, (e) backstroke, and (f) exit. The first round workshop (with TFG) resulted in a consensus for test procedure fundamentals (see Table 2), including characteristics of high proficiency levels for each aquatic skill. In round two, consensus was reached on four proficiency levels for each aquatic skill, ranging from very high to very low levels of mastery, based on a critical revision of the first draft of SCAS. In the final part of the Delphi study (round three), a national independent expert panel rated the relevance, representativeness, and clarity of the instrument for measuring swimming competence in children, through an online survey. Based on interpretations of the responses in a content validity index framework (Lynn, 1986; Polit & Beck, 2006), results indicated high item (range: 80–93%) and overall scale agreement (88%) among independent experts in evaluating the content validity of SCAS. Additionally, we conducted a small pilot study on children (*n* = 12), resulting in minor adjustments to the instructions in the test protocol.

It is important to emphasize that SCAS can be relevant for researchers, teachers, and instructors working within swimming and aquatic education contexts. From a research perspective, the current study contributes to operationalizing the Norwegian standard on swimming competence. Additionally, the SCAS can capture variations in aquatic skill levels, ranging from very high to very low, enabling a valid screening of baseline swimming competence from individual to population levels. The World Health Organization (2021) has stated that empowering children with aquatic skills is an important intervention for safe engagement in water environments. Both knowledge and providing statistics about swimming competence at a population level, including children and youth, is a vital component to address in a national drowning prevention strategy. Indeed, Button (2016) explained that basic aquatic skills represent a platform for the learning of aquatic motor skills, and a generic building block for more complex skills required for prevention of fatal and non-fatal drowning accidents. The SCAS can inform and contribute to the longitudinal mapping of aquatic skills across time, and influence the long journey towards being water competent, as well as supporting the development of pedagogical models in learn-to-swim programs. At an individual level, the SCAS holds the potential to explore children’s aquatic learning at an early stage, including examining the strengths and weaknesses of their aquatic capabilities.

The utility of an assessment instrument for the teaching profession, or for others responsible for in-school learn-to-swim programs, is embedded in their formative assessment and mapping strategies. Formative assessment is grounded to a statutory requirement in the regulation to the Educational Act, which includes an interaction between the individual, the environment and the task, and it must be an integrated part of the educational training (Ministry of Education and Research, 2021). Bergene et al. (2022) reported that 76% of the Norwegian primary schools conducted a swimming proficiency test in the 2021-2022 schoolyear. Based on our Delphi study, we suggest that the SCAS is a valid and objective assessment instrument to be used as the compulsory swimming proficiency test, and further, it can be applied towards screening an initial level of proficiency. Furthermore, the SCAS can be utilized to evaluate the effectiveness of current learn-to-swim programs and for tracking pupils’ attainments of the swimming competence objectives in the PE curriculum. In this context, the SCAS differs from other published measurement instruments, due to the comprehensiveness and continuous assessment of aquatic skills on a four-point scale that describes various levels. In a school context, the SCAS can help identify learning difficulties and challenges (e.g., children with lower mastery levels in specific aquatic skills) and it can support the child’s learning and development by redirecting resources or time spent on learning. The SCAS can support the school-home communication with parents and caregivers regarding the pupil’s competency status, and this may enhance awareness of their actual swimming proficiency and encourage parents to help their children practice needed aquatic skills.

### Limitations and Future Directions

Various water environments such as lakes, rivers or the ocean largely contain the same characteristics around the globe (e.g., density or currents). This implies that basic aquatic skills are a generic matter, and that the SCAS contains an evaluation of key aspects within swimming and water competence that can have international applicability. The water competence concept embodies a holistic and dynamic approach to drowning prevention that integrates psychomotor tasks, cognitive knowledge and affective attitudes (Langendorfer et al., 2018). Arguably, the SCAS represents the psychomotor aspects of the water competence model that consist of seven skills proposed by Stallman et al. (2017): (a) safe entry competency, (b) breath control competency, (c) stationary surface competency, (d) water orientation competency, (e) propulsion competency, (f) underwater competency, and (g) safe exit competency. However, it is important to recognize that SCAS does not capture the entire water competence model, due to little attention to cognitive and affective aspects, that are vital for reflecting the complexity of drowning prevention situations. Although the SCAS is designed and developed in an indoor swimming pool context, we propose that the component skills related to water competence are influenced by conditions of the aquatic environment (e.g., variations in water temperature, clarity, depth or distance) and specific task demands (e.g., variations in clothing and/or equipment), that an individual may be introduced to. An international group of drowning prevention experts has emphasized through a consensus-based process the importance of learn-to-swim and water safety survival skills to promote recreational open water drowning prevention (Quan et al., 2012). The degree to which an individual’s aquatic behaviors in a swimming pool compare with the greater complexity of various environmental conditions or task demands must be further explored. Moreover, SCAS can contribute to the theoretical development of the water competence model by investigating and exploring the transfer of aquatic skills in various water environments.

One of the major purposes of this study was to design and develop a valid assessment instrument that operationalizes the curricular competence aim on swimming competence. The study involved 22 participants with diverse aquatic expertise, and a modified Delphi approach served as the framework for structuring the development and to explore the community of opinions throughout the process. The starting point for this study differed from a common Delphi approach (e.g., a traditional iterative polls) as the current study had to rely on a pre-defined scope provided by the specific competence aim in the curriculum as well as the objective towards developing an assessment instrument. Modifications to the Delphi study had to be implemented to fit the study purpose and provide an adequate structure that facilitated communication and solicited opinions in working toward a shared interpretation of the content. However, this study still fit within the Delphi inquiry designs toolkit (Day & Bobeva, 2005). The design refers to a rich variety of Delphi applications as brainstorming, focus groups and questionnaires, to discuss and vote on specific topics. In response to the objective of SCAS being a resource for both researchers and practitioners, a heterogeneous group of participants were recruited. In the initial stage of the process, a majority of practitioners with extensive experience in the field were central, whilst the researcher’s point of view was integrated in the later rounds for content validation. The deliberate management of participants’ roles and an uneven number of participants in subgroups did not appear to influence the outcome of developing SCAS, as it maintained high consensus among TFG and IEP in how to conduct and measure the proficiency test, and level of competence among children in the six aquatic skills. However, a Delphi study highly depends on the ability and qualifications of the selected panel of experts, and is not necessarily repeatable with other groups (Bulger & Housner, 2007).

At this point, the psychometric properties of the SCAS need to be assessed in addition to the content validity reported in this article. In the future, estimating consistency over time (test-retest reliability) and observational ratings provided by multiple coders (inter-rater reliability) should be evaluated. In addition, a further validation of the construct is recommended and is currently under investigation.

## Conclusion

Based upon the presented Delphi study and corresponding process, the main take home messages from this study are that the SCAS developed in this process can now be used to observe and assess basic aquatic skills according to learning objectives in the fourth grade of the Norwegian PE curriculum for primary schools. Additionally, the SCAS has utility regarding the psychomotor aspect of the water competence model. The instrument can strengthen pedagogical development in learn-to-swim programs as it can be a source for screening, evaluation, and formative assessment. Furthermore, important data can be collected for research and development purposes to enhance theoretical and methodological development of this tool and to further educational work related to swimming and water competence.

## Supplemental Material

Supplemental Material - Development and Content Validation of the Swimming Competence Assessment Scale (SCAS): A Modified Delphi Study Click here for additional data file. Supplemental Material for Development and Content Validation of the Swimming Competence Assessment Scale (SCAS): A Modified Delphi Study Jon Sundan, Monika Haga, Håvard Lorås in Perceptual and Motor Skills.

## References

[bibr1-00315125231177403] Bergene A. C. Solbue K. Lynnebakke B. Ramberg I. Wollscheid S. (2022). Spørsmål til Skole-Norge - Analyser og resultater fra Utdanningsdirektoratets spørreundersøkelse til skoler og skoleeiere våren 2022. [Diractorate of Education`s survey of schools and school owners]. https://www.udir.no/tall-og-forskning/finn-forskning/rapporter/sporsmal-til-skole-norge-varen-2022/

[bibr2-00315125231177403] Bierens J. J. L. M. (2014). Drowning: Prevention, rescue, treatment (2nd ed.). Springer.

[bibr3-00315125231177403] Bratten J. H. Kilanowska J. (2021). Physical education and new forms of activity following the implementation of the core curriculum in Norway in 2020. Journal of Education, Health and Sport, 11(12), 56–68. 10.12775/JEHS.2021.11.12.005

[bibr4-00315125231177403] Brenner R. A. Saluja G. Smith G. S. (2003). Swimming lessons, swimming ability, and the risk of drowning. Injury Control and Safety Promotion, 10(4), 211–216. 10.1076/icsp.10.4.211.16775 14664364

[bibr5-00315125231177403] Bulger S. M. Housner L. D. (2007). Modified delphi investigation of exercise science in physical education teacher education. Journal of Teaching in Physical Education, 26(1), 57–80. 10.1123/jtpe.26.1.57 25141085

[bibr6-00315125231177403] Button C. (2016). Aquatic locomotion: Forgotten fundamental movement skills? Journal of New Zealand Physical Educator, 49(1), 8–10. 10.3316/informit.163744995642658

[bibr7-00315125231177403] Button C. Button A. J. Jackson A.-M. Cotter J. D. Maraj B. (2020). Teaching foundational aquatic skills to children in open water environments. International Journal of Aquatic Research and Education, 13(1), 1–24. 10.25035/IJARE.13.01.01

[bibr8-00315125231177403] Day J. Bobeva M. (2005). A generic tookit for the successful management of Delphi studies. Electronic Journal of Business Research Methods, 3(2), 103–116. https://academic-publishing.org/index.php/ejbrm/article/view/1195/1158

[bibr9-00315125231177403] Department for Education . (2013). The national curriculum in England: Key stages 1 and 2 framework document. https://www.gov.uk/government/publications/national-curriculum-in-england-primary-curriculum

[bibr10-00315125231177403] Diamond I. R. Grant R. C. Feldman B. M. Pencharz P. B. Ling S. C. Moore A. M. Wales P. W. (2014). Defining consensus: A systematic review recommends methodologic criteria for reporting of delphi studies. Journal of Clinical Epidemiology, 67(4), 401–409. 10.1016/j.jclinepi.2013.12.002 24581294

[bibr11-00315125231177403] Erbaugh S. J. (1978). Assessment of swimming performance of preschool children. Perceptual and Motor Skills, 47(3 Pt 2), 1179–1182. 10.2466/pms.1978.46.3f.1179 745893

[bibr12-00315125231177403] Finish Swimming TeachingAssociation, L. (2011). Kuudesluokkalaisten ja aikuisten uimataito Suomessa vuonna 2011. [Swimming skills of sixth graders and adults in Finland in 2011. https://www.suh.fi/files/200/uimataitoraportti_fin.pdf

[bibr13-00315125231177403] Jünger S. Payne S. A. Brine J. Radbruch L. Brearley S. G. (2017). Guidance on Conducting and REporting DElphi Studies (CREDES) in palliative care: Recommendations based on a methodological systematic review. Palliative Medicine, 31(8), 684–706. 10.1177/0269216317690685 28190381

[bibr14-00315125231177403] Kjendlie P.-L. Pedersen T. Thoresen T. Setlo T. Moran K. Stallman R. K. (2013). Can you swim in waves? Children's swimming, floating, and entry skills in calm and simulated unsteady water conditions. International Journal of Aquatic Research and Education, 7(4), 301–313. 10.25035/ijare.07.04.04

[bibr15-00315125231177403] Langendorfer S. Bruya L. (1995). Aquatic Readiness: Developing water comptence in young children. Human Kinetics.

[bibr16-00315125231177403] Langendorfer S. Moran K. Stallman R. (2018). Guiding principles: Applying water competence to drowning prevention. International Journal of Aquatic Research and Education, 11(2), 1–3. 10.25035/ijare.11.02.22

[bibr17-00315125231177403] Linstone H. A. Turoff M. (1975). The Delphi method: Techniques and applications. Addison-Wesley.

[bibr18-00315125231177403] Lynn M. R. (1986). Determination and quantification of content validity. Nursing Research, 35(6), 382–386. 10.1097/00006199-198611000-00017 3640358

[bibr19-00315125231177403] Mertens L. De Martelaer K. Sääkslahti A. D’hondt E. (2021). The inter-rater and intra-rater reliability of the actual aquatic skills test (Aast) for assessing young children’s motor competence in the water. International Journal of Environmental Research and Public Health, 19(1), 446. 10.3390/ijerph19010446 35010700 PMC8744731

[bibr20-00315125231177403] Ministry of Education and Research . (2021). Regulation to the educational Act (FOR-2006-06-23-724). Lovdata. https://lovdata.no/dokument/SF/forskrift/2006-06-23-724

[bibr21-00315125231177403] Ministry of Church and Education . (1957). Normalplan for byfolkeskolen (1939). [National core curriculum 1939]. Aschehoug. https://www.nb.no/nbsok/nb/a772fcd5e1bbbfb3dcb3b7e43d6ccc60?index=1#1

[bibr22-00315125231177403] Moran K. (2013). Defining ‘swim and survive’ in the context of New Zealand drowning prevention strategies: A discussion paper. https://www.dpanz.org.nz/wp-content/uploads/2019/06/Water-competency-in-the-context-of-New-Zealand-drowning-prevention-strategies-Kevin-Moran-120713.pdf

[bibr23-00315125231177403] Mordal Moen K. Westlie K. Bjørke L. Brattli V. H. (2018). Når ambisjon møter tradisjon - En nasjonal kartleggingsstudie av kroppsøvingsfaget i grunnskolen (5-10. trinn). [When ambition meets tradition -A national study of physical education in primary school (grade 5-10)] https://brage.inn.no/inn-xmlui/handle/11250/2482450.

[bibr24-00315125231177403] Moreno-Murcia J. A. (2005). Desarrollo y validación preliminar de escalas para la evaluación de la competencia motriz acuática en escolares de 4 a 11 años. (Development and preliminary validation of an aquatic competence scale for children 4 to 11 years old.). RICYDE. Revista internacional de ciencias del deporte, 1(1), 14–27. 10.5232/ricyde2005.00102

[bibr25-00315125231177403] Moreno-Murcia J. A. Borges L. d. P. Hernández E. H. (2020). Design and validation of the scale to measure aquatic competence in children (SMACC). International Journal of Environmental Research and Public Health, 17(17), 1–16. 10.3390/ijerph17176188 PMC7503350 32858995

[bibr26-00315125231177403] Norwegian Directorate for Education and Training . (2020). Læreplan i kroppsøving (KRO01-05). Established as regulations. The national curriculum for the Knowlegde promotion 2020. https://www.udir.no/lk20/kro01-05

[bibr27-00315125231177403] Norwegian Swimming Federation . (2021). Undersøkelser om svømmedyktighet blant elever i 5. klasse. [Survey of swimming ability among students in 5th grade]. https://svomming.no/nyheter/undersokelser-om-svommedyktighet

[bibr28-00315125231177403] Polit D. F. Beck C. T. (2006). The content validity index: Are you sure you know what's being reported? Critique and recommendations. Research in Nursing and Health, 29(5), 489–497. 10.1002/nur.20147 16977646

[bibr29-00315125231177403] Quan L. Bennett E. Moran K. Bierens J. J. L. M. (2012). Use of a consensus-based process to develop international guidelines to decrease recreational open water drowning deaths. International Journal of Health Promotion and Education, 50(3), 135–144. 10.1080/14635240.2012.661968

[bibr30-00315125231177403] Quan L. Ramos W. Harvey C. Kublick L. Langendorfer S. Lees T. A. Fielding R. R. Dalke S. Barry C. Shook S. Wernicki P. (2015). Toward defining water competency: An American Red Cross definition. International Journal of Aquatic Research and Education, 9(1), 12–23. 10.1123/ijare.2014-0066

[bibr31-00315125231177403] Ross S. Metcalf A. Bulger S. M. Housner L. D. (2014). Modified Delphi investigation of motor development and learning in physical education teacher education. Research Quarterly for Exercise and Sport, 85(3), 316–329. 10.1080/02701367.2014.930087 25141085

[bibr32-00315125231177403] Royal Life Saving Society Australia (2019). National swimming and water safety framework. https://www.royallifesaving.com.au/educate-participate/swimming/national-swimming-and-water-safety-framework

[bibr33-00315125231177403] Stallman R. K. (2017). From swimming skill to water competence: A paradigm shift. International Journal of Aquatic Research and Education, 10(2), 1–5. 10.25035/ijare.10.02.02

[bibr34-00315125231177403] Stallman R. K. Junge M. Blixt T. (2008). The teaching of swimming based on a model derived from the causes of drowning. International Journal of Aquatic Research and Education, 2(4), 372–382. 10.25035/ijare.02.04.11

[bibr35-00315125231177403] Stallman R. K. Moran K. Quan L. Langendorfer S. (2017). From swimming skill to water competence: Towards a more inclusive drowning prevention future. International Journal of Aquatic Research and Education, 10(2). 10.25035/ijare.10.02.03

[bibr36-00315125231177403] United Nations Educational Scientific and Cultural Organization (2014). World-wide survey of school physical education: Final report 2013 https://unesdoc.unesco.org/ark:/48223/pf0000229335

[bibr37-00315125231177403] Vernon W. (2009). The delphi technique: A review. International Journal of Therapy and Rehabilitation, 16(2), 69–76. 10.12968/ijtr.2009.16.2.38892

[bibr38-00315125231177403] Wizer T. Cássia Daniele Zaleski T. Wellington Gomes F. Dayana da Silva O. Flávio Antônio de Sorza C. (2021). Assessment instruments for children in the aquatic environment: A systematic review. Motricidade, 17(3), 1–37. 10.6063/motricidade.21586

[bibr39-00315125231177403] World Health Organization . (2014). Global report on drowning: Preventing a leading killer. https://www.who.int/publications/i/item/global-report-on-drowning-preventing-a-leading-killer

[bibr40-00315125231177403] World Health Organization . (2017). Preventing drowning: An implementation guide. https://www.who.int/publications/i/item/9789241511933

[bibr41-00315125231177403] World Health Organization . (2021). WHO Guideline on the prevention of drowning through provision of day-care and basic swimming and water safety skills. https://www.who.int/publications/i/item/9789240030008 34379372

[bibr42-00315125231177403] World Health Organization . (2022). Preventing drowning: Practical guidance for the provision of day-care, basic swimming and water safety skills, and safe rescue and resuscitation training. https://www.who.int/publications/i/item/9789240046726

[bibr43-00315125231177403] Yusoff M. S. B. (2019). ABC of content validation and content validity index calculation. Education in Medicine Journal, 11(2), 49–54. 10.21315/eimj2019.11.2.6

