# Supplementary material for: Development and Content Validation of the Swimming Competence Assessment Scale (SCAS): A Modified Delphi Study
Source: Percept Mot Skills. 2023 May 18;130(4):1762–80. doi: 10.1177/00315125231177403 (PMC10363955; doi:10.1177/00315125231177403)
Supplement: Supplemental Material - Development and Content Validation of the Swimming Competence Assessment Scale (SCAS): A Modified Delphi Study [file sj-pdf-1-pms-10.1177_00315125231177403.pdf]

NTNU
Norwegian University of Science and Technology

Swimming Competence Assessment Scale (SCAS)
OBSERVATION FORM

Category	Score		Description
Entry	4	Excellent	Fall into deep water fully submersed, resurface and continues
	3	High	Fall into deep water from squat position fully submersed, resurfaces and continues
	2	Low	Fall into deep water immersed or submersed, resurfaces, holds on to edge/swim lane lines or similar before continuing
	1	Poor	Unable to fall into deep water and submersion, and enter by climbing
Frontstroke	4	Excellent	Swimming continuously 100 meters frontstroke
	3	High	Swimming 100 meters frontstroke, but has to stop and rest/float in the water along the way
	2	Low	Swimming 100 meters frontstroke, but has to rest one or more times standing on the pool floor, hanging on the edge, or pool lane lines
	1	Poor	Unable to swim 100 meters frontstroke
Surface dive	4	Excellent	Dives from the surface to the pool floor, and perform the task on first attempt
	3	High	Dives from the surface to the pool floor, and perform the task on second attempt
	2	Low	Dives from the surface to the pool floor, but need three or more attempts to perform the task
	1	Poor	Unable to surface dive to the pool floor and perform the task
Float / Rest	4	Excellent	Floats effortlessly for 3 minutes without correction resting position significantly
	3	High	Floats relatively effortlessly for 3 minutes, but corrects resting position repeatedly with active movements
	2	Low	Floats strained for 3 minutes, and must work hard to be able to hold the resting position
	1	Poor	Unable to float for 3 minutes
Backstroke	4	Excellent	Swimming continuously 100 meters backstroke
	3	High	Swimming 100 meters backstroke, but has to stop and rest/float in the water along the way
	2	Low	Swimming 100 meters backstroke, but has to rest one or more times standing on the pool floor, hanging on the edge, or pool lane lines
	1	Poor	Unable to swim 100 meters backstroke
Exit	4	Excellent	Exits the pool to an edge elevated above the water surface
	3	High	Exits the pool to an edge horizontally with the water surface
	2	Low	Exits the pool to an edge horizontally with the water surface, but needs several attempts
	1	Poor	Unable exit the pool without using a ladder/stair or other assistance

NTNU
Norwegian University of Science and Technology

NTNU
Norwegian University of
Science and Technology

Swimming Competence Assessment Scale (SCAS)
CODING SHEET

Municipality		School		Date	
Length swimming pool		Depth swimming pool		Height elevated edge	

Student	Entry				Surface dive				Frontstroke				Backstroke				Float				Exit				SC*
First name / Last name	1	2	3	4	1	2	3	4	1	2	3	4	1	2	3	4	1	2	3	4	1	2	3	4	

* = Swimming competent. Mark if student is considered as a competent swimmer (X) or not (-)
Test not completed: Mark with **N-C** behind student’s name
Exempt implementation: Mark with **E-I** behind student’s name

NTNU
Norwegian University of
Science and Technology

Swimming Competence Assessment Scale (SCAS)
PROCEDURES FOR SWIMMING PROFICIENCY TEST

About the test:

- Before the test, students shall receive information about what they are going to do, and test manager verbally goes through how the test will be conducted.
- Vigorous physical activity right before the test should be avoided.
- The test should be conducted in regular swimming clothes (shorts, bathing suit, or similar clothing)
- Swimming goggles can be used if necessary.
- Teachers/instructors should not apply pressure to the student during the test, but encouraged through positive feedback and comments.

Conducting the test:

- **Entry**: The student stands at the edge of the swimming pool and start the test by falling into the water, before resurfacing and continues. Elaboration:
  - Falling into the water is not the same as jumping into the water. The student can enter sideways, backwards, provided a soft push to simulate an unintentional fall into water.
  - Falling into deep water must be conducted in deep water, and the body shall be submerged no matter what entry method used.
- **Frontstroke**: The student swims 100 meters frontstroke across the swimming pool. Elaboration:
  - Swimming stroke on front is optional, and no focus on time spent or technical execution
  - Excellent score (4) demands that the student shall not touch the bottom, the wall, or the swimming lane lines with the purpose of resting
  - Touching the bottom/swimming lane lines by accident or unintentionally should not be included in the evaluation.
  - If the student fails to swim 100 meters continuously, encourage to rest or float (instead of resting at the bottom/edge/swimming lane lines) and then continue
- **Surface dive**: The student will dive from the surface and perform a task. Elaboration:
  - The surface dive task is to retrieve a ring (or another object) with the hands.
  - Diving depth of approximately 1.3 meters. Minimum depth is where the student cannot stand at the bottom.
  - The student may choose their preferable diving technique from the surface (not by jumping). Both with head first (horizontal to vertical) or feet first are approved techniques, as long as object is collected with their hands.
- **Backstroke**: The student swims 100 meters backstroke across the swimming pool
  - When all students are swimming on their backs, the test manager will blow a whistle.
  - Same descriptions as for frontstroke.
- **Floating**: Float and rest stationary in the water for 3 minutes
  - The students start by floating on their front and then turn to their backs when desired.
  - When the student is finished floating, they may continue to swim backstroke until they have covered 100 meters.
- **Exit**: Climb ashore from the pool without using the swim lane lines, diving board or similar
  - Elevated edge is the level difference from the water surface to the edge (approximately 30 cm above the water surface)
  - If the student does not manage to climb the elevated edge, they may try to exit from a edge horizontal to the surface.
  - The test ends when the student exits the swimming pool.
- **Transitions:**
  - The students should have the opportunity to show their level of mastery in all six aquatic skills. If someone does not master one of the aquatic skills, they should be able to move on to the next.
  - There’s no need to rush the transitions, the students may use some time to think about how to solve the upcoming task.
  - With the exception of entry and exit, the order of the aquatic skills can be changed.
